# Supplementary material for: Structure-guided discovery of anti-CRISPR and anti-phage defense proteins
Source: Nat Commun. 2024 Jan 20;15:649. doi: 10.1038/s41467-024-45068-7 (PMC10799925; doi:10.1038/s41467-024-45068-7)
Supplement: Supplementary file 1 — Supplementary Information [file 41467_2024_45068_MOESM1_ESM.pdf]

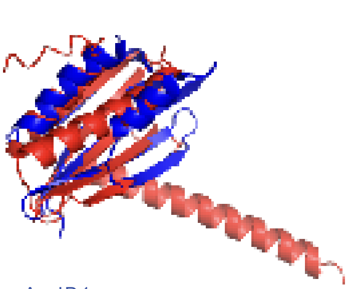

AcrID1
IMGVR_UViG_3300005530_000034|-
Ga0070679_100000205|-
Ga0070679_1000002053
TM-score=0.6249

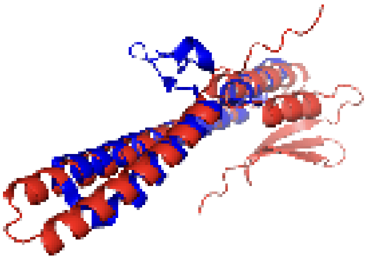

AcrIE1
IMGVR_UViG_3300022214_000006|-
Ga0224505_10000123|-
Ga0224505_1000012393
TM-score=0.6648

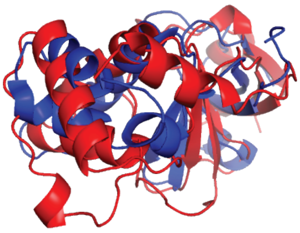

AcrIF11
IMGVR_UViG_3300031813_000024|-
Ga0316217_10000462|-
Ga0316217_1000046230
TM-score=0.6372

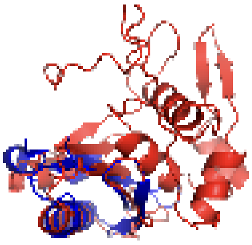

AcrIF5
IMGVR_UViG_3300033174_000053|-
Ga0334882_1003602|-
Ga0334882_100360223
TM-score=0.6902

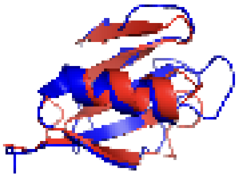

AcrIF9
IMGVR_UViG_3300009370_000194|-
Ga0118716_1002994|-
Ga0118716_100299430
TM-score=0.7027

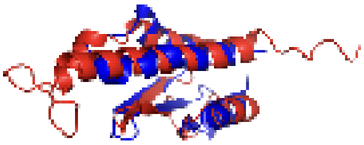

AcrIIA4
UGV-GENOME-0360355|UGV-GE-
NOME-0360355_19
TM-score=0.6444

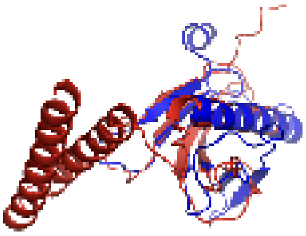

AcrIIC2
UGV-GENOME-0368711|UGV-GE-
NOME-0368711_46
TM-score=0.6017

Supplementary Figure 1: Structural comparison between putative and known Acr proteins. AlphaFold2 model of candidate Acrs (in red) superposed to known Acr proteins (blue). The candidate Acrs are indicated by their IMG/VR gene id.

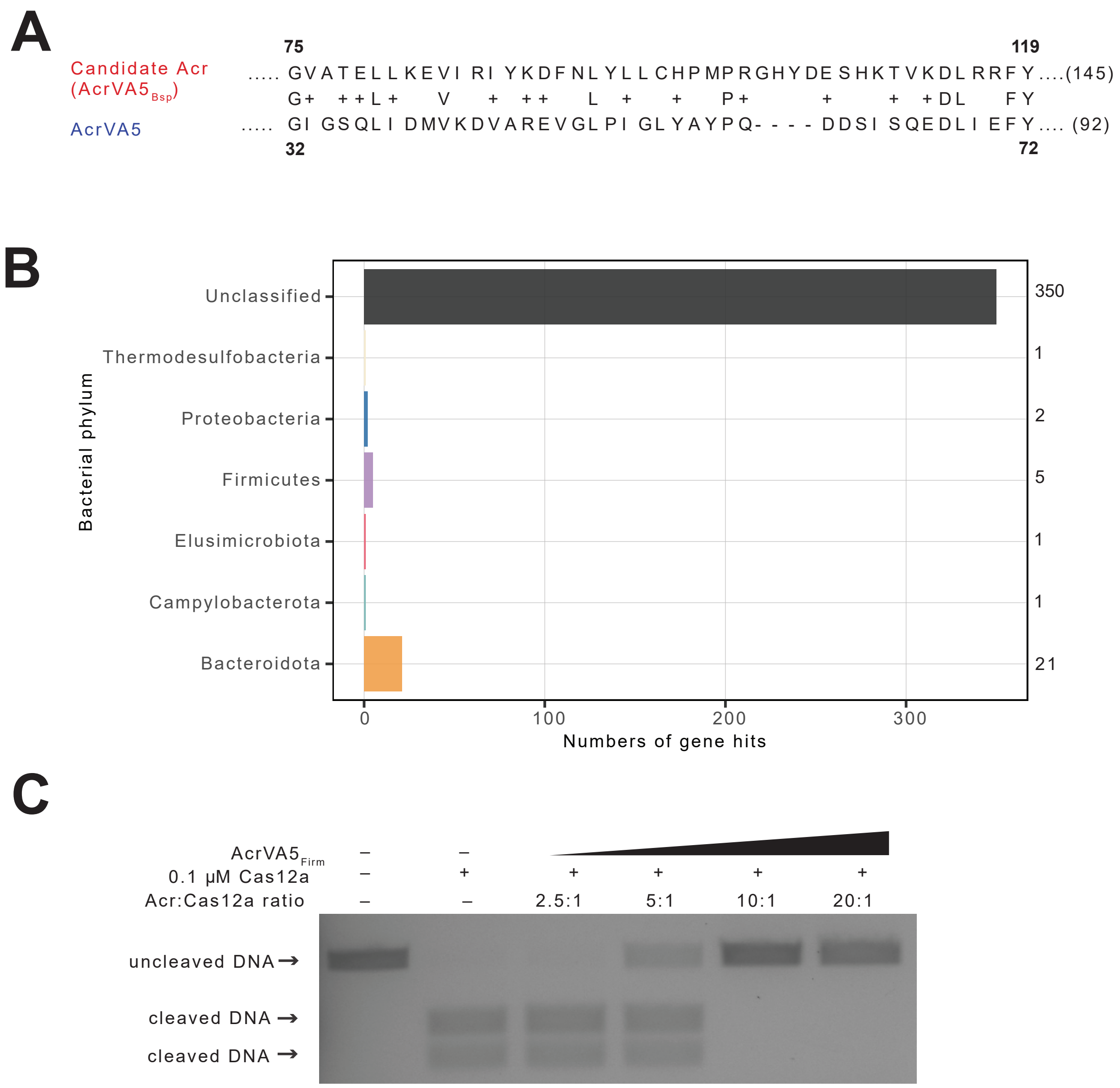

Supplementary Figure 2: Characterization of AcrVA5$_{Bsp}$ and homolog from a Firmicutes host. (A) Primary sequence alignment between AcrVA5 and AcrVA5$_{Bsp}$. The numbers in parenthesis show the protein length, while the numbers in bold represent the alignment start and end positions. No significant similarity between both proteins at an evalue threshold of 0.05. (B) Distribution of BLAST hits of protein-coding genes in the scaffold containing AcrVA5$_{Bsp}$. The BLAST search was implemented in the online server of IMG/VR. Though most of the genes were unassigned, 21 genes had hits in a *Bacteroidota* genome. (C) Agarose gel showing LbCas12a-mediated dsDNA cleavage in the presence of increasing amounts of AcrVA5$_{Firm}$. Acr concentrations used are 0.25, 0.5, 1, and 2 μM. Image is representative of duplicate. Source data are provided as a Source Data file.

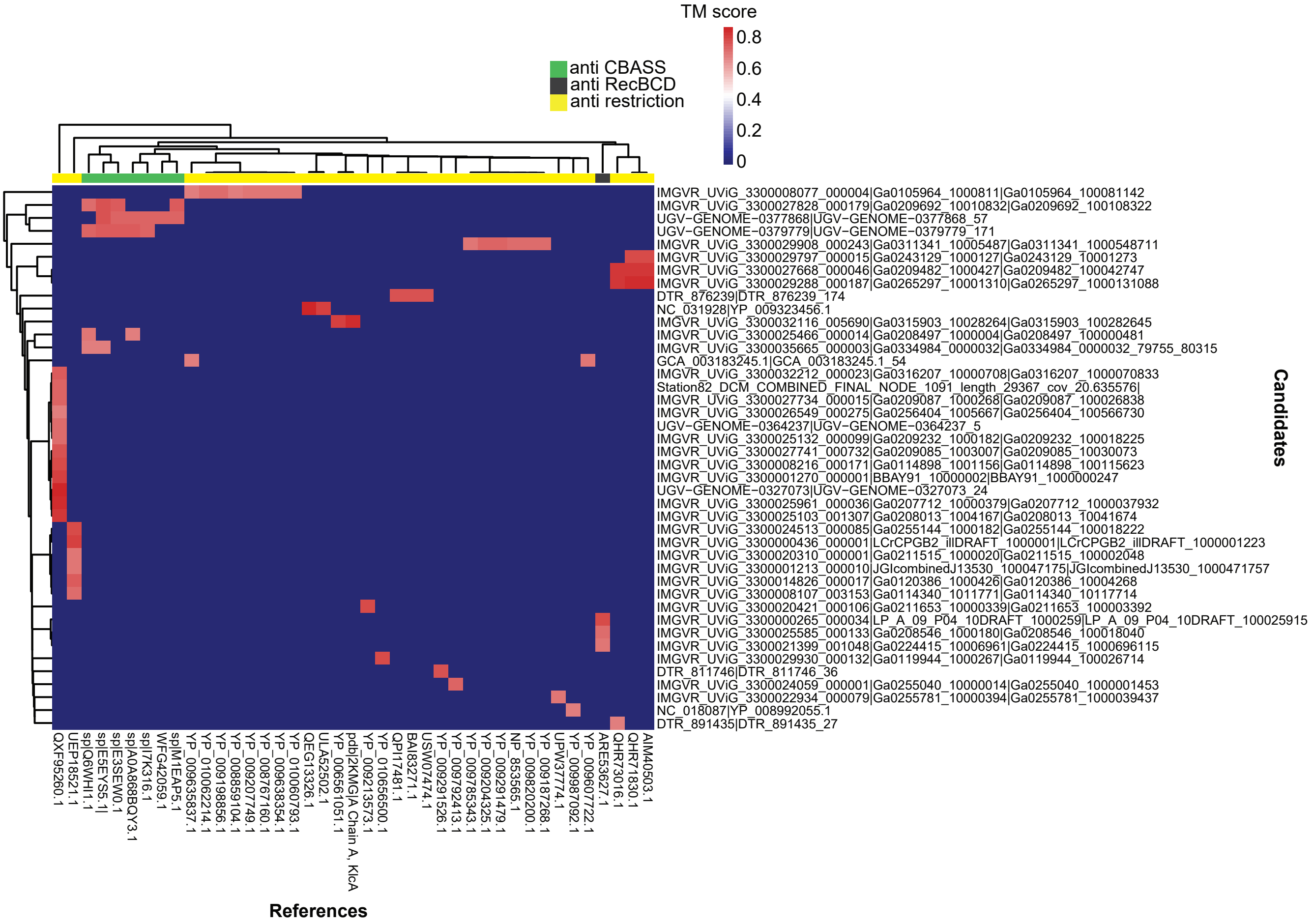

Supplementary Figure 3: Identification of remote homologs of other phage counter-defenses. Heatmap showing proteins in IMG/VR (rows) with no sequence similarity, but having high structural similarities to known counter-defenses (columns). For instance, DTR_891435|DTR_891435_27 is structurally similar to a known anti-restriction protein (QHR73016.1)

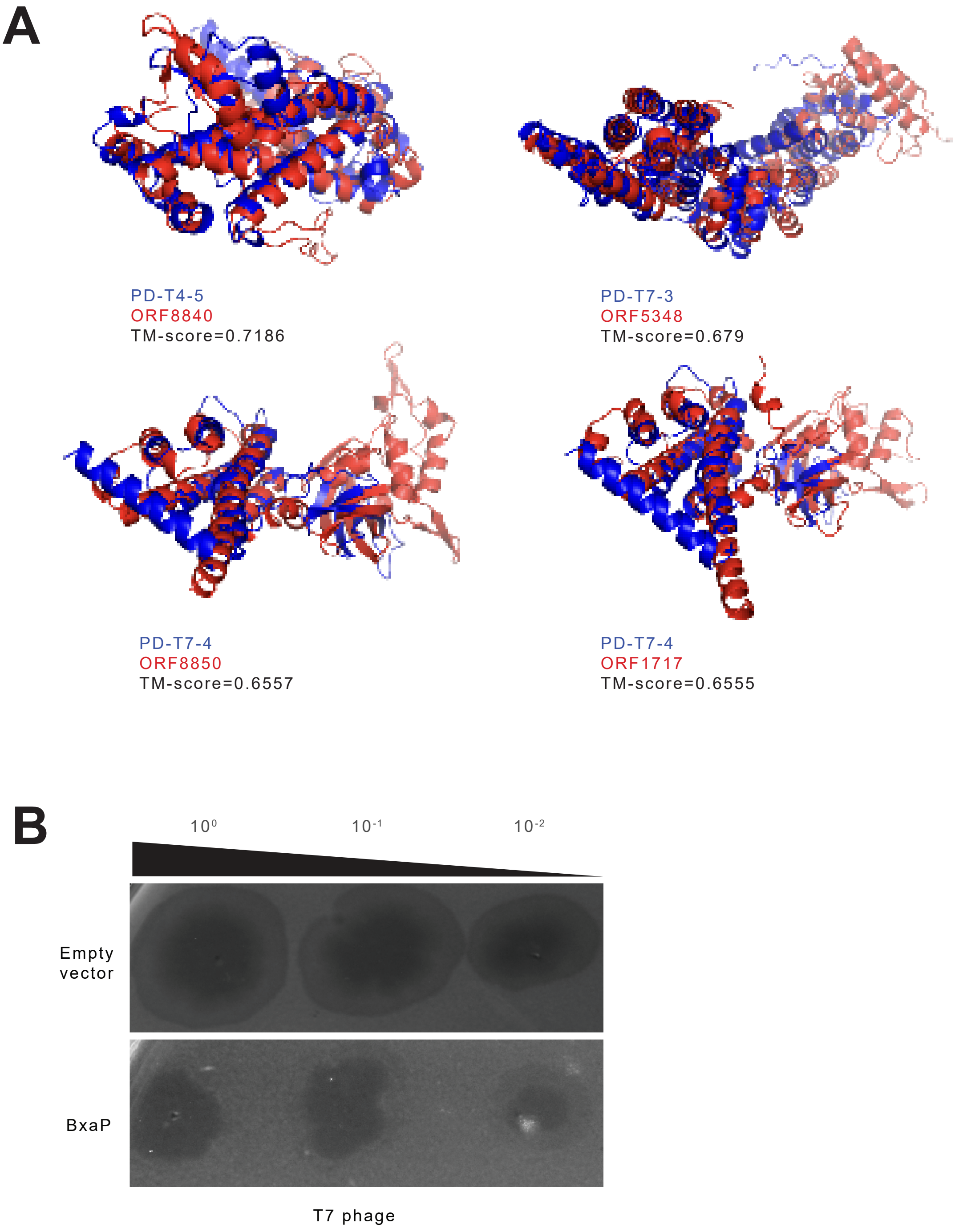

Supplementary Figure 4: Structural similarities between putative and known defense proteins and effect of BxaP expression on T7 infection.
(A) AlphaFold2 model of candidate defense proteins (in red) superposed to predicted single-protein defense systems reported in Vassallo *et al.* (blue). (B) A 10-fold serial dilution plaque assay of T7 phage on *E. coli* strain containing either an empty vector or overexpressing BxaP. Image is representative of triplicate.

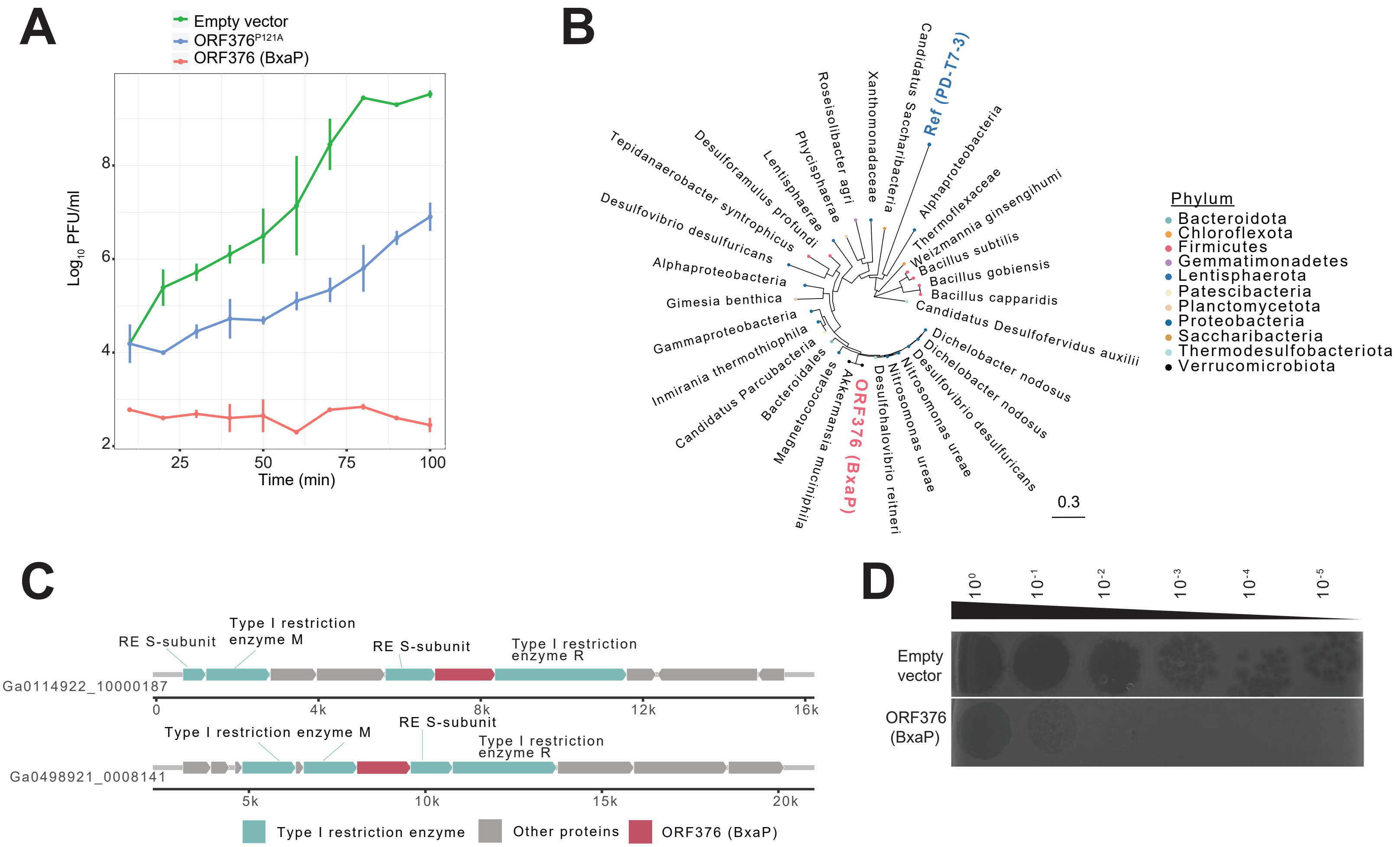

Supplementary Figure 5: BxaP protects against phage infection.
(A) One-step growth curves showing measurements of plaque forming units (PFU) over time in T4-infected *E. coli* strain containing either an empty vector, or overexpressing WT or mutant BxaP. Experiments were conducted in duplicates, and error bars represent SEM. (B) Phylogenetic distribution of BxaP homologs in NCBI non-redundant database. Branch tips are colored based on bacteria phyla. PD-T7-3 is included in the tree for reference purposes. (C) Examples of phage genomic loci containing BxaP. The IMG/VR ID are shown beside each scaffold. (D) A 10-fold serial dilution plaque assay of T4 phage on an *E. coli* strain (DH10B) lacking both BREX and type-I RM systems. Image is representative of duplicate. Source data are provided as a Source Data file.

```
Bxap                                                                    ----------------------MVATVDLSHYSEQIPAAEDRPLFDEAVDTGKAGALRATFVMIWLACAESLKRRFREAQK-RDSTA
Reference PD-T7-3                                                       ----------------------MDVRIFSLESQKSKIYDRRTRKYFEEVYKSYANGCYRSATVMLWSVVVCDIIFKLQELRDVHNDAV
WP 227119521.1 hypothetical protein Desulfovibrio desulfuricans         ----------------------MTAPVDLSHYADNILAAEDRPLFDDAVEAGKAGALRAAYVMIWLACAESLKRRFREAQK-RDGAA
WP 029894982.1 hypothetical protein Desulfohalovibrio reitneri          ----------------------MSAPVDLSHYADNILAAEDRPLFDDAVESGKAGALRAAYVMIWLACAESLKRRFREAQK-RDGAA
SNX61233.1 hypothetical protein SAMN06296273 2685 Nitrosomonas ureae   ----------------------MSAPVDLSHYADNILAAEDRPLFDDAVESGKAGALRAAYVMIWLACAESLKRRFREAQK-RDGAA
WP 172424124.1 hypothetical protein Nitrosomonas ureae                  -------------------------------------------------------------------------------------
WP 211086234.1 hypothetical protein Bacillus capparidis                 --------------------------MDFNVLRGQIVEDRDKVLFDEAVNCLNGNALRAAYITTWISTAESLKLKFYDMSS-RDHEI
WP 053605790.1 hypothetical protein Bacillus gobiensis                  MLPDKKMYFQFLIRYLLINFSNSEEEDMDFNVLRGQIVEDRDKVLFDEAVNCLNGNALRAAYITTWISTAESLKLKFYDMSS-RDHEI
MBR5129867.1 hypothetical protein Alphaproteobacteria bacterium        ------------------------MGFDIS----KIKNKEDRALISDAIKLCENGFYRASYIMAWLSCAESLKRRFYELGK-RDSNA
RMH17673.1 hypothetical protein D6698 08045 Gammaproteobacteria bacterium  MSEPIQEPNVQPVDLSNYSAAILYSEDRPLFDDAVAAAKAGALRAAYVMIWLACAESLKRRFREAQK-RDSAA

                                                                                                                                             121
Bxap                                                                    -GKIVGGIATKEKAHQAVDKF---VLTKACEYG-FISDSEYTVLNHIYEMRCLYGHPYEE------APSEEQVIHAAAMVVEHVLSRP
Reference PD-T7-3                                                       AEKILLEIEALQNDDPYSPKWEKELIKRVFERTQLLDTASNHKVLLIQKHRHLSAHPVISDEDTLFEPTQEMIRSDIRNSIEVILSKP
WP 227119521.1 hypothetical protein Desulfovibrio desulfuricans         -GKIVGEIETKEKEHKAVDKF---VLMKAHEYG-FVSDSGHTVLNHIYEMRCLYGHPYEE------APSQEQVSHAAAVVVEHVLSKP
WP 029894982.1 hypothetical protein Desulfohalovibrio reitneri          -GKIVGEIETKEKEHKAVDKF---VLMKAHEYG-FVSDSGHTVLNHIYEMRCLYGHPYEE------APSQEQVSHAAAVVVEHVLSKP
SNX61233.1 hypothetical protein SAMN06296273 2685 Nitrosomonas ureae   -GKIVGEIETKEKEHKAVDKF---VLMKAHEYG-FVSDSGHTVLNHIYEMRCLYGHPYEE------APSQEQVSHAAAVVVEHVLSKP
WP 172424124.1 hypothetical protein Nitrosomonas ureae                  ----------------------------------------------MRCLYGHPYEE------APSQEQVSHAAAVVVEHVLSKP
WP 211086234.1 hypothetical protein Bacillus capparidis                 KKKVIGKIEDLEQKQRPTDSF---LIRSAKDFG-LISAEQSKKLEHMKDMRGVYAHPLNA------APSDSEVELAIELSVNIVLSQP
WP 053605790.1 hypothetical protein Bacillus gobiensis                  KKKVIGKIEDLEQKQRPTDSF---LIRSAKDFG-LISAEQSKKLEHMKDMRGVYAHPLNA------APSDSEVELAIELSVNIVLSQP
MBR5129867.1 hypothetical protein Alphaproteobacteria bacterium        -GKLYKNIEELEKQHKSVDNE---IINGAFNFN-LISDTEKEKLKHFFSMRSVYSHPYEE------APSKVDCEQIIENIIQIVLSRP
RMH17673.1 hypothetical protein D6698 08045 Gammaproteobacteria bacterium  -GSIVGDIETKEREHRSVDKF---VLDKAHEYG-FLSDPGHTILNHVYEMRCLYGHPYEE------APSQEQVSHAAAVVVEHVLSKP

Bxap                                                                    VKLRHGFGKQLLTNLLGSPNFLDDQQTAVLAFTKDILPRLDDDIYGWLLDNYWG-----ELEKISDDSSMAIFFRRGTWFSCTMLTEI
Reference PD-T7-3                                                       PFMSQKILSTFVADLEKVKDLFPSDNALKKYLDVKYFKSL NKEVLVKIFKGLWKFSFRSEEAKPLENREINI---RAMKLIFEKDRQA
WP 227119521.1 hypothetical protein Desulfovibrio desulfuricans         VKLRHGFGKQLLKSLLEEPNFLDDQQTAVVAFTKDILPRLDESIHGWLLDNYWE-----ELEKFSDDSSMAIFFRRGTWFSRTMLTEV
WP 029894982.1 hypothetical protein Desulfohalovibrio reitneri          VKLRHGFGKQLLKSLLEEPNFLDDQQTAVVAFTKDILPRLDETIHGWLLDNYWA-----ELEKFSDDSSMAIFFRRGTWFSRTMLTEV
SNX61233.1 hypothetical protein SAMN06296273 2685 Nitrosomonas ureae   VKLRHGFGKQLLKSLLEEPNFLDDQQTAVVAFTKDILPRLDETIHGWLLDNYWA-----ELEKFSDDSSMAIFFRRGTWFSRTMLIEV
WP 172424124.1 hypothetical protein Nitrosomonas ureae                  VKLRHGFGKQLLKSLLEEPNFLDDQQTAVVAFTKDILPRLDETIHGWLLDNYWA-----ELEKFSDDSSMAIFFRRGTWFSRTMLIEV
WP 211086234.1 hypothetical protein Bacillus capparidis                 ALLKHAFVQSLVTSIFENHHYLDDSTETVQKAAISTLNHVHSSVFPYFFKLLIE-----NLNKVSKDFTKDLFERRGSVFL-DMLLKC
WP 053605790.1 hypothetical protein Bacillus gobiensis                  ALLKHAFVQSLVTSIFENHHYLDDSTETVQKAAISTLNHVHSSVFPYFFKLLIE-----NLNKVSKDFTKDLFERRGSVFL-DMLLKC
MBR5129867.1 hypothetical protein Alphaproteobacteria bacterium        VLLKEGGISFILDRLTKEESFLNNSSTEINTYSKELISIIDPKKYSYFLKKYLQ-----FLNDTPKDQRKGILYNRGIMILSYMAFNI
RMH17673.1 hypothetical protein D6698 08045 Gammaproteobacteria bacterium  VKLRHGFGKQLLKSLLENRNYLDDQQTAVTAFTKDIIPRLDESIHGWLLDKYWE-----ELEELADDSSMAIFFRRGIWFCRTMLTEV

Bxap                                                                    GID- VFSHDDWH--DRLSKFP---KILMRVC-SIADIFKEIGDRAQDSLVGLIIA---ESATRA----SVLTYLEQLIINGALTKRQR
Reference PD-T7-3                                                       MVDSVKAETAYY--SNISNNHDAIKALIEFISMEKEIYNALDDSVKE-LIKPIIK------------DNISYFGIAFFISESPEEHI
WP 227119521.1 hypothetical protein Desulfovibrio desulfuricans         GID-VFSHDDWH--DRSSRFP---KILMRVC-SIADIFKEIGKRAQDSLVGLIIA---ESATRA----   SVLTHLERLSINGALTMRQQ
WP 029894982.1 hypothetical protein Desulfohalovibrio reitneri          GID-VFSHDDWH--DRSSRFP---KILMRVC-SIADIFKEIGKRAQDSLVGLIIA---ESATRA----   SVLTHLERLSINGALTTRQQ
SNX61233.1 hypothetical protein SAMN06296273 2685 Nitrosomonas ureae   GID-VFSHDDWH--DRSSRFP---KILMRVC-SIADIFKEIGKRAQDSLVGLIIA---EAATRA----   SVLTHLERLSINGALTTRQQ
WP 172424124.1 hypothetical protein Nitrosomonas ureae                  GID-VFSHDDWH--DRSSRFP---KILMRVC-   SIADIFKEIGKRAQDSLVGLIIA---EAATRA----SVLTHLERLSINGALTTRQQ
WP 211086234.1 hypothetical protein Bacillus capparidis                 SLK-NFSTESWSLEGLLHKYP---SIITKVF-   VKEDYWTHLNDILKDAMIGHLIEPIGDDGEVETPSLENLTSVLNLYNQDLLNERHV
WP 053605790.1 hypothetical protein Bacillus gobiensis                  SLK-NFSTESWSLEGLLHKYP---SIITKVF-   VKEDYWTHLNDILKDAMIGHLIEPIGDDGEVETPSLENLTSVLNLYNQDLLNERHV
MBR5129867.1 hypothetical protein Alphaproteobacteria bacterium        GIENIWSNEEFE--NILYTYR---NASLNVF-AELNIFLLLDQKFKNIIINRLFE---DIPDNL----   SLIKYLDSFYIKNILTENQR
RMH17673.1 hypothetical protein D6698 08045 Gammaproteobacteria bacterium  GVD-VFSHDEWH--ERAGRFP---KTLMRVC-GIADIFKEIGNRAQDALVGSILV---ESSTHA----   SVLTYLERLNNDGALSVRQQ

Bxap                                                                    ERF-----------VECVSE--MSLD-----LLMSAGLSTKTCYRKLIDAMKSHNWYLQNPAIDIIVSNGPEQAADLDEKQQENLGRN
Reference PD-T7-3                                                       NRV-----------TKRISEKYYKKYGDNGNFLNQQHLAI---   FKNVCSELGLESEYRDFGIACFINSADFER--------------
WP 227119521.1 hypothetical protein Desulfovibrio desulfuricans         ERF-----------VEHVSE--MPSS-----AIRSAGLSTKTCYGKLIDAMKSHNWYVQNPAIDLIVSNGPDQAAELDENQQVNLGRN
WP 029894982.1 hypothetical protein Desulfohalovibrio reitneri          ERF-----------VEHVSE--MPSS-----AIRSAGLSTKTCYGKLIDAMKSHNWYVQNPAIDLIVSNGPDQAAELDENQQVNLGRN
SNX61233.1 hypothetical protein SAMN06296273 2685 Nitrosomonas ureae   ERF-----------VEHISE--MPSS-----AIRSAGLSTKTCYGKLIDAMKSHNWYVQNPAIDLIVSNGPDQAAELDENQQVNLGRN
WP 172424124.1 hypothetical protein Nitrosomonas ureae                  ERF-----------VEHISE--MPSS-----AIRSAGLSTKTCYGKLIDAMKSHNWYVQNPAIDLIVSNGPDQAAELDENQQVNLGRN
WP 211086234.1 hypothetical protein Bacillus capparidis                 ERF-----------KSAVEK--LYIS-----KKVSIGIPLEWYQAELISDLKTSNWYAQNPVIEAIQSLGVEKINTLDSEFLIELGRN
WP 053605790.1 hypothetical protein Bacillus gobiensis                  ERF-----------KSAVEK--LYIS-----KKVSIGIPLEWYQAELISDLKTSNWYAQNPVIEAIQSLGVEKINTLDSEFLIELGRN
MBR5129867.1 hypothetical protein Alphaproteobacteria bacterium        RKF-----------SFIVFD--INWR-----DLPSIELSLKALYPRIISGLKSHNWHMQNPACEYIIHFGSFD-DTLSDEEYVELGRN
RMH17673.1 hypothetical protein D6698 08045 Gammaproteobacteria bacterium  ERF-----------TERISE--MDSS-----ELRSAALSTKTCYEKLIAAMKSYDWYVQNPAIELIVSNGPDQAAELTEQQQIELGRN

Bxap                                                                    LLQAGEGSAASADEFL-   EKLSQ-DKIS--WPFPVISGIAMES-FTNENNQIRFKKRHWERVLSIID-HLPQELQGRLIHQISESVDAG
Reference PD-T7-3                                                       -----------ADIYF-DRFIDKDLAN--YSSEQMLTLLEGA---NKNNQCYWRNR-----------SRNGNDSIRILKAAKNKLPDG
WP 227119521.1 hypothetical protein Desulfovibrio desulfuricans         LLQAGEGTAGSANEFL-EKLSQ-DGTS--WPFHVVRGIAMES-FTNEDNQIRFKDRHLGRVLSAID-   HLQQELQDQLIAEISASVDAG
WP 029894982.1 hypothetical protein Desulfohalovibrio reitneri          LLQAGEGTAGSANEFL-EKLSQ-DGTS--WPFHVVRGIAMES-FTNEDNQIRFKDRHLGRVLAAID-   HLQQELQDQLIAELSASVDAG
SNX61233.1 hypothetical protein SAMN06296273 2685 Nitrosomonas ureae   LLQAGEGTAGSANEFL-EKLSQ-DGTS--WPFHVVRGIAMES-FTNEDNQIRFKDRHLGRVLAAID-   HLQQELQDQLMAELSASVDAG
WP 172424124.1 hypothetical protein Nitrosomonas ureae                  LLQAGEGTAGSANEFL-EKLSQ-DGTS--WPFHVVRGIAMES-   FTNEDNQIRFKDRHLGRVLAAID-HLQQELQDQLMAELSASVDAG
WP 211086234.1 hypothetical protein Bacillus capparidis                 ILQAADGQARDAISFV-SSLYK-SEFK--SSPYLVEGIFLET-   FINENKRFRFK-RYRKPALSAVL-CVNEQETDKIIDKAIMLLKDC
WP 053605790.1 hypothetical protein Bacillus gobiensis                  ILQAADGQARDAISFV-SSLYK-SEFK--SSPYLVEGIFLET-   FINENKRFRFK-RYRKPALSAVL-CVNEQETDKIIDKAIMLLKDC
MBR5129867.1 hypothetical protein Alphaproteobacteria bacterium        ILQSYVGGSSGAHSLL-YRIST-VGN---TPISLIQGIVEEC-   FYDDNKKLRLKISNPQLIEQIIDKNLSFEEQENLFLEIATNIQQT
RMH17673.1 hypothetical protein D6698 08045 Gammaproteobacteria bacterium  ILQSAEGASNMAARFL-VRLSD-EGSR--WPLGIVRGIALET-FTNEKQELRFKTKHLHRVLDALD-   RLPNTELEQVVAEVSESIVKS

Bxap                                                                    APK-Y-L--GGRDNFRNVIDSL-----------NAYSWATPLLESL-EAKSAEILTEEEEVWPLKS----------------------   500
Reference PD-T7-3                                                       ------F------DFSKYDNLP------------------------                                              455
WP 227119521.1 hypothetical protein Desulfovibrio desulfuricans         VPK-D-W--VDRDDFENAVDSL-----------KAYPWAAPLVTSL-EAKAASLSAEEEDA---------------------------    495
WP 029894982.1 hypothetical protein Desulfohalovibrio reitneri          APK-D-W--VDRDDFESAVESL-----------NAYAWAAPLVASL-EAKSASLSAEEEDA---------------------------    495
SNX61233.1 hypothetical protein SAMN06296273 2685 Nitrosomonas ureae   TPK-D-W--VDRDDFESAVESL-----------NAYAWAAPLVASL-EAKSASLSAEEEDA---------------------------    495
WP 172424124.1 hypothetical protein Nitrosomonas ureae                  TPK-D-W--VDRDDFESAVESL-----------NAYAWAAPLVASL-EAKSASLSAEEEDA---------------------------    387
WP 211086234.1 hypothetical protein Bacillus capparidis                 SLR-G-W--FNEETFEEYLEYLDEILNFKKLKGLKKEAFEKFNLVF-KETCDRFKQEEEEEELEV-----------------------   514
WP 053605790.1 hypothetical protein Bacillus gobiensis                  SLR-G-W--FNEETFEEYLEYLDEILNFKKLKGLKKEAFEKFNLVF-KETCDRFKQEEEEEELEV-----------------------   541
MBR5129867.1 hypothetical protein Alphaproteobacteria bacterium        EIKRN-W---DWSEYDQIPDTG---------------------L-LGKIKKVLNENYPVFPF------------------------   480
RMH17673.1 hypothetical protein D6698 08045 Gammaproteobacteria bacterium  EPQ-F-W--VSREELGDVIDSL-----------KPFSWASALISSL-ETKAGEFTGEDEET---------------------------   503
```

Supplementary Figure 6: Primary sequence alignment of some BxaP homologs. The shaded area shows mutated site used to generate the P121A BxaP mutant.
