# Supplementary material for: Structure-guided discovery of anti-CRISPR and anti-phage defense proteins
Source: Nat Commun. 2024 Jan 20;15:649. doi: 10.1038/s41467-024-45068-7 (PMC10799925; doi:10.1038/s41467-024-45068-7)
Supplement: Supplementary file 3 — Description of Additional Supplementary Files [file 41467_2024_45068_MOESM3_ESM.pdf]

Description of Additional Supplementary Files:

Supplementary Data 1: Published Acrs and their properties.

Supplementary Data 2: Comparison between putative Acrs and published Acrs.

Supplementary Data 3: Performance of Acr prediction tools with AcrVA5Bsp.

Supplementary Data 4: Putative Acrs structurally homologous to two known Acrs.

Supplementary Data 5: Comparison between putative anti-phage proteins and published defense systems.

Supplementary Data 6: Nucleic acid sequences used in this study.

Supplementary Data 7: Bacteria strain and plasmids used in this study.
