# Supplementary material for: Structure-guided discovery of anti-CRISPR and anti-phage defense proteins
Source: Nat Commun. 2024 Jan 20;15:649. doi: 10.1038/s41467-024-45068-7 (PMC10799925; doi:10.1038/s41467-024-45068-7)
Supplement: Supplementary file 5 — Reporting Summary [file 41467_2024_45068_MOESM5_ESM.pdf]

nature portfolio

Corresponding author(s): Akintunde Emiola

Last updated by author(s): Dec 19, 2023

Reporting Summary

Nature Portfolio wishes to improve the reproducibility of the work that we publish. This form provides structure for consistency and transparency in reporting. For further information on Nature Portfolio policies, see our Editorial Policies and the Editorial Policy Checklist.

Statistics

For all statistical analyses, confirm that the following items are present in the figure legend, table legend, main text, or Methods section.

n/a	Confirmed	
☐	☒	The exact sample size (*n*) for each experimental group/condition, given as a discrete number and unit of measurement
☐	☒	A statement on whether measurements were taken from distinct samples or whether the same sample was measured repeatedly
☐	☒	The statistical test(s) used AND whether they are one- or two-sided<br>*Only common tests should be described solely by name; describe more complex techniques in the Methods section.*
☒	☐	A description of all covariates tested
☐	☒	A description of any assumptions or corrections, such as tests of normality and adjustment for multiple comparisons
☐	☒	A full description of the statistical parameters including central tendency (e.g. means) or other basic estimates (e.g. regression coefficient) AND variation (e.g. standard deviation) or associated estimates of uncertainty (e.g. confidence intervals)
☐	☒	For null hypothesis testing, the test statistic (e.g. *F*, *t*, *r*) with confidence intervals, effect sizes, degrees of freedom and *P* value noted<br>*Give P values as exact values whenever suitable.*
☒	☐	For Bayesian analysis, information on the choice of priors and Markov chain Monte Carlo settings
☒	☐	For hierarchical and complex designs, identification of the appropriate level for tests and full reporting of outcomes
☒	☐	Estimates of effect sizes (e.g. Cohen's *d*, Pearson's *r*), indicating how they were calculated

Our web collection on statistics for biologists contains articles on many of the points above.

Software and code

Policy information about availability of computer code

Data collection	BLAST v. 12.13.0 for homology searches, USEARCH v. 11.0.667 for protein clustering, and PADLOC v. 1.1.0 for predicting known defense systems
Data analysis	Alphafold2 v. 2.1.2 was used to predict protein structures, US-align v. 20220511 for pairwise structure alignment, IPC v. 2.0 to determine isoelectric point (pI) values of proteins, MUSCLE v. 5.1 for multiple sequence alignment, and FastTree v. 2.1.11 for generation of phylogenetic tree. PyMOL (v. 2.6.0) was used to visualized superposed structures. Furthermore, the online version of HHpred was used to detect remote structural similarities to known protein domains/families. Lastly, we developed a tool to retrieve uncharacterized genes between known anti-phage defense genes. The software is freely available at https://github.com/EmiolaLab/ExtractGenes

For manuscripts utilizing custom algorithms or software that are central to the research but not yet described in published literature, software must be made available to editors and reviewers. We strongly encourage code deposition in a community repository (e.g. GitHub). See the Nature Portfolio guidelines for submitting code & software for further information.

Data

Policy information about availability of data

All manuscripts must include a data availability statement. This statement should provide the following information, where applicable:

- Accession codes, unique identifiers, or web links for publicly available datasets
- A description of any restrictions on data availability
- For clinical datasets or third party data, please ensure that the statement adheres to our policy

Phage protein sequences were retrieved from IMG/VR (https://img.jgi.doe.gov/cgi-bin/vr/main.cgi) while MAGs were retrieved from GEM (https://genome.jgi.doe.gov/portal/GEMs/GEMs.home.html) and GMBC (https://gmgc.embl.de/download.cgi). The ~300,000 predicted protein structures in this study can be downloaded from https://figshare.com/articles/dataset/Duan_protein_structures2024/24948714. All other necessary data are included in the Supplementary Information. Source data are provided with this paper.

Research involving human participants, their data, or biological material

Policy information about studies with human participants or human data. See also policy information about sex, gender (identity/presentation), and sexual orientation and race, ethnicity and racism.

Reporting on sex and gender	N/A
Reporting on race, ethnicity, or other socially relevant groupings	N/A
Population characteristics	N/A
Recruitment	N/A
Ethics oversight	N/A

Note that full information on the approval of the study protocol must also be provided in the manuscript.

Field-specific reporting

Please select the one below that is the best fit for your research. If you are not sure, read the appropriate sections before making your selection.

[x] Life sciences [ ] Behavioural & social sciences [ ] Ecological, evolutionary & environmental sciences

For a reference copy of the document with all sections, see nature.com/documents/nr-reporting-summary-flat.pdf

Life sciences study design

All studies must disclose on these points even when the disclosure is negative.

Sample size	For phage analysis, we randomly selected 285,000 proteins for structure prediction from our catalog of ~7 million proteins. Previous studies have identified putative Acrs by analyzing fewer phage proteins. Predicting structures for only a subset was necessary because of the huge computational constraint associated with such large dataset.
Data exclusions	No data was excluded
Replication	All experiments were conducted in triplicates and successfully reproduced
Randomization	Randomization was not necessary because the control and experimental groups were done in isogenic strains
Blinding	Blinding was not possible as the individual performing the experiments typically prepared the samples being measured

Reporting for specific materials, systems and methods

We require information from authors about some types of materials, experimental systems and methods used in many studies. Here, indicate whether each material, system or method listed is relevant to your study. If you are not sure if a list item applies to your research, read the appropriate section before selecting a response.

Materials & experimental systems

n/a	Involved in the study
☒	☐ Antibodies
☒	☐ Eukaryotic cell lines
☒	☐ Palaeontology and archaeology
☒	☐ Animals and other organisms
☒	☐ Clinical data
☒	☐ Dual use research of concern
☒	☐ Plants

Methods

n/a	Involved in the study
☒	☐ ChIP-seq
☒	☐ Flow cytometry
☒	☐ MRI-based neuroimaging

Plants

Seed stocks	N/A
Novel plant genotypes	N/A
Authentication	N/A
